# CIGS thin-film solar module processing: case of high-speed laser scribing

**DOI:** 10.1038/srep40502

**Published:** 2017-01-13

**Authors:** Paulius Gečys, Edgaras Markauskas, Shiro Nishiwaki, Stephan Buecheler, Ronny De Loor, Andreas Burn, Valerio Romano, Gediminas Račiukaitis

**Affiliations:** 1 Center for Physical Sciences and Technology, Savanoriu Ave. 231, LT-02300, Vilnius, Lithuania; 2 Laboratory for Thin Films and Photovoltaics, Swiss Federal Laboratories for Materials Science and Technology EMPA, Ueberlandstrasse 129, CH-8600 Duebendorf, Switzerland; 3 Next Scan Technology, Noorwegenstraat 29, 9940 Evergem, Belgium; 4 Bern University of Applied Sciences, Pestalozzistrasse 20, CH-3400 Burgdorf, Switzerland

## Abstract

In this paper, we investigate the laser processing of the CIGS thin-film solar cells in the case of the high-speed regime. The modern ultra-short pulsed laser was used exhibiting the pulse repetition rate of 1 MHz. Two main P3 scribing approaches were investigated – ablation of the full layer stack to expose the molybdenum back-contact, and removal of the front-contact only. The scribe quality was evaluated by SEM together with EDS spectrometer followed by electrical measurements. We also modelled the electrical behavior of a device at the mini-module scale taking into account the laser-induced damage. We demonstrated, that high-speed process at high laser pulse repetition rate induced thermal damage to the cell. However, the top-contact layer lift-off processing enabled us to reach 1.7 m/s scribing speed with a minimal device degradation. Also, we demonstrated the P3 processing in the ultra-high speed regime, where the scribing speed of 50 m/s was obtained. Finally, selected laser processes were tested in the case of mini-module scribing. Overall, we conclude, that the top-contact layer lift-off processing is the only reliable solution for high-speed P3 laser scribing, which can be implemented in the future terawatt-scale photovoltaic production facilities.

Cu(In,Ga)Se_2_ thin-film solar cell technology has a great potential for low-cost, high-performance solar panel production. The reports of record-breaking efficiencies appear every year, making this technology the most efficient one among the thin-film based solar cells. The record value of 22.6% stands for the CIGS fabricated on a rigid glass substrate^1^, while 20.4% was obtained on flexible polyimide substrate^2^. Usually, the record performance is demonstrated on small-area devices, therefore, upscaling the high-efficiency device over the large area is crucial for the future development of the CIGS technology. Commercial production of these devices still faces serious challenges in terms of preserving the cell efficiency at the module scale, production throughput, process reliability, and costs. Moving towards large-scale production, innovative technological solutions are required in the manufacturing process.

Laser-scribed monolithic interconnects are one of the key processes in maintaining the module efficiency. The module sized CIGS device needs to be divided into smaller cells interconnected in series. This way the photo-current is limited, reducing the resistive losses in thin layers^3^. Usually, three laser scribing steps called P1, P2 and P3 are used for the module processing. P1 patterns the back-contact forming the stripe-shaped molybdenum grid; P2 is used for the series interconnect formation between the adjacent cells after the CIGS deposition; the P3 process is used for the neighboring cell isolation after the top-contact deposition. The P3 process is the most challenging one since laser-induced thermal effects can lead to CIGS structural changes^4^,^5^, the formation of electro-conductive secondary phase^5^, and degradation of device electrical properties^6^,^7^.

Various laser sources have been tested for the P2 and P3 type laser scribing, although ultra-short pulsed laser has been reported as the most suitable tool^5^,^7^,^8^,^9^. In the case of the third isolating scribe (P3), two main scribing approaches were reported in the literature – ablation of the full layer stack to expose the molybdenum back-contact^5^,^10^,^11^, and the removal of the front-contact only^9^,^11^,^12^. These two methods can be addressed as the laser scribe P3 “type 1” and “type 2”, respectively. In the case of the P3 “type 1” scribing, the material removal process is direct material ablation. Therefore, the ultra-short laser pulses are used to optimize the scribing quality. In the case of the P3 “type 2” processing, the top-contact is removed by the laser-induced lift-off process reducing remaining thermal effects. However, the underneath CIGS layer can be affected as well, causing shunting of the device. Fine tuning of the laser process parameters can help to minimize undesirable absorber layer modifications for both “type 1“ and “type 2“ processes, but thermal effects are still inevitable.

Industrial scale implementation of the laser scribing technology requires a sufficient process throughput. In other words, the linear scribing speed exceeding 2 m/s is required. The most of the previous research was based on ultra-short pulsed lasers operating at low pulse repetition rates (≤200 kHz)^8^,^9^,^13^ setting the P2 and P3 “type 1” process scribing speed limit below 300 mm/s. Generally, it was possible to increase the scribing speed by applying low pulse overlap process, unfortunately, with a compromise in the process quality.

In this paper, we investigate the laser processing of the CIGS thin-film solar cells in the case of the high-speed regime. Modern ultra-short lasers can offer high average powers at high pulse repetition rates (≥1 MHz). Therefore, it is possible to upscale the scribing speed maintaining the optimal pulse overlap. Unfortunately, the high pulse repetition rate could lead to heat accumulation effects^14^,^15^, potentially lowering the scribe quality. Therefore, it is necessary to validate the laser scribing processes at high pulse repetition rate conditions.

## Results

### Laser scribing experiment

**Figure 1:**
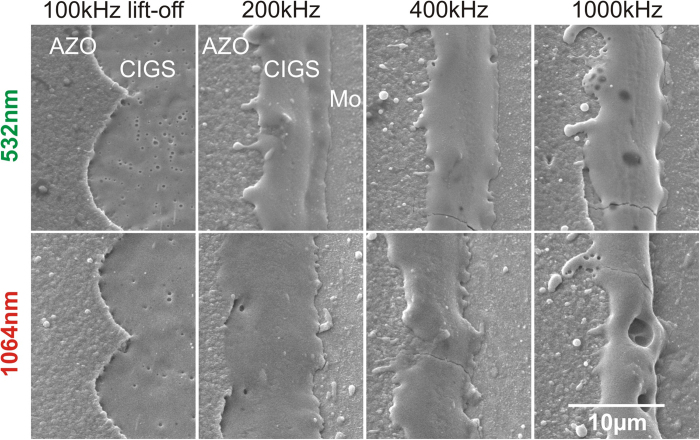
SEM images of the P3 channel edges. Scribing was performed at variuos laser pulse repetition rates keeping the optimal pulse overlap. The 100** **kHz processing represents the P3 “type 2” pattern, while P3 “type 1” processing was made with 200–1000** **kHz laser repetition rate.

Laser scribing tests were performed on CIGS solar cell samples. Two main laser scribing approaches of the P3 process were investigated – removal of the CIGS and Al:ZnO (AZO) layers to expose the Mo back-contact (P3 “type 1”), and the removal of the front-contact only (P3 “type 2”). Laser scribing parameters are presented in Table 1. In the case of the P3 “type 1” process, the laser pulse repetition rate and scribing speed were varied, keeping the optimal pulse overlap of 91–92%. During the P3 “type 2” processing, the laser repetition rate of 100 kHz with pulse overlap of 23% was used pushing the scribing speed to the limit of 1.7 m/s of the used galvanometer scanner. SEM images of the laser processed channels are shown in Fig. 1. The width of the P3 scribes was in the range of 30 μm. In the case of direct ablation using 1064 nm wavelength, thermal modification of the CIGS was observed even for the 200 kHz laser processing. However, the thermal effects were further enlarged when switching to the 400–1000 kHz range. Also, cracking of the P3 channel edge was more pronounced in this case. Switching to the 532 nm wavelength did not introduce any significant changes despite that the laser fluence was reduced from 3.8 J/cm^2^ to 2.9 J/cm^2^. Thermal modification and cracking of the scribe edge was present and was enlarged with an increase of the process speed.

Ultra-short pulsed lasers are usually associated with “cold ablation”, resulting in a high-quality material removal mechanism with minimized thermal effects. However, the high pulse repetition rate processing can lead to heat accumulation effects lowering the process quality^14^,^16^. In our case, the laser pulse repetition rate of 1 MHz was sufficient enough to introduce heat-related effects.

In the case of the P3 “type 2” processing, the AZO layer lift-off process was introduced. The laser fluence and pulse overlap were lowered to 1.6–1.7 J/cm^2^ and 23%, respectively. It helped to produce crack-free scribes with relatively low damage to the surrounding CIGS material (see Fig. 1). There was no significant difference in terms of the process quality for the 1064 nm/532 nm wavelength processing, although only visual quality (SEM images) was compared. Since relatively low laser pulse repetition rate was used, no heat accumulation effects were observed in this case (see Fig. 1).

### Scribe electrical testing

Cu-chalcopyrite is a complex multi-component compound, and laser thermal modification can lead to structural disorders of the material and secondary phase formation near the ablation area^4^,^5^. Furthermore, laser-induced heating could result in a partial vaporization of the CIGS composing elements forming Cu-rich compound^17^. All these laser-induced changes can form short-circuit in the cell. Therefore, measurement of the scribe specific conductance is an important step for the process quality control.

**Figure 2:**
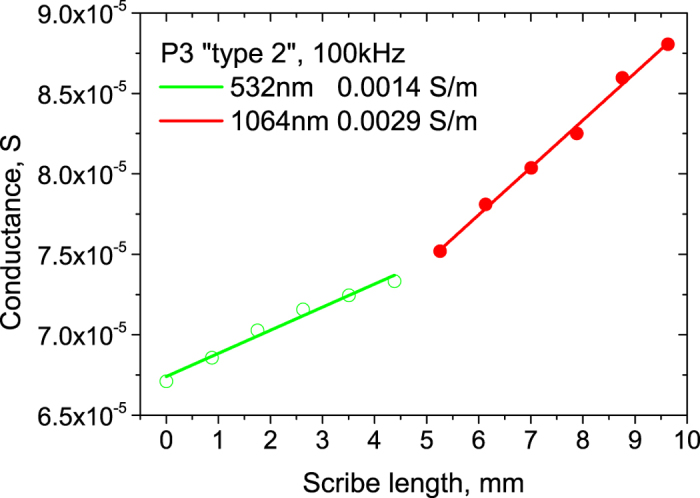
Parallel conductance of the cell vs. length of the laser scribe. The P3 “type 2” scribe conductivity measurement at the same cell area were performed for both laser wavelengths. The pulse repetition rate 100** **kHz, scribing speed 1.7** **m/s: (red line) laser wavelength - 1064** **nm, fluence - 1.7** **J/cm^2^; (green line) laser wavelength - 532** **nm, fluence - 1.6** **J/cm^2^.

In order to evaluate the scribe quality in terms of electrical behavior, the sequence of 0.7 mm-long P3 scribes was performed, followed by the cell parallel conductance measurement after each scribe (LLST method). Afterwards, the scribe specific conductance was extracted from the measurement by fitting the data with a simple linear function (see Fig. 2). In the case of the P3 “type 2” processing, the tests were performed on the same sample area without contact probe removal during the entire experiment including processing with both 1064 nm and 532 nm wavelengths. That enabled us to eliminate measurement errors caused by contacting and material properties variation. The measurements are presented in Fig. 2. The scribe specific conductance of 0.0029 S/m was obtained for the P3 “type 2” 1064 nm wavelength processing. However, the laser lift-off processing with the 532 nm wavelength showed two times lower scribe specific conductance of 0.0014 S/m. In this case, the longer wavelength induced more damage during the scribing. That resulted changes in the cell electrical behavior, although the scribe visual examination with SEM did not reveal any visual differences (see Fig. 1).

**Figure 3:**
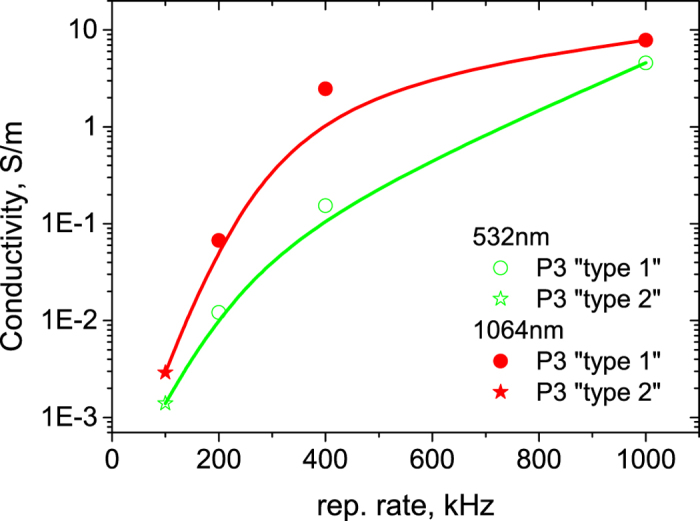
Laser scribe conductivity vs. laser repetition rate. The measurements were performed for both 1064** **nm and 532** **nm wavelength processing.

In the case of the P3 “type 1” 1064 nm wavelength processing, the scribe specific conductance was measured for all processing conditions that are presented in Table 1. A significant increase of the scribe specific conductance was observed when switching from the layer lift-off to direct ablation regime. Even laser processing with relatively low pulse repetition rate of 200 kHz induced severe changes in the cell’s electrical behavior (see Fig. 3). Further increase of the scribing speed and laser pulse repetition rate resulted in even more damage to the cell (see Fig. 3). Finally, the high-speed laser processing with 1 MHz pulse repetition rate and fundamental harmonics resulted in the scribe specific conductance of 7.9 S/m, completely short-circuiting the cell. Processing with the 532 nm wavelength helped to reduce the scribe conductivity to 4.6 S/m, although the changes were not significant (see Fig. 3).

### Serial interconnect simulation

**Figure 4:**
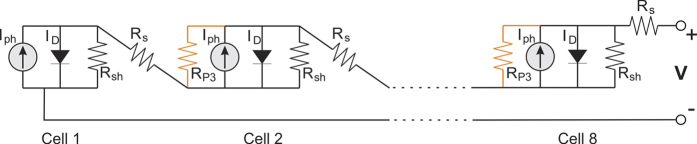
Equivalent circuit of a 8-cell mini-module interconnected in series.

We conducted a simple CIGS solar cell mini-module simulation to predict behavior of the laser scribed module efficiency. For such task, we used a PSpice modelling software that is widely used in many photovoltaic simulation tasks ranging from a single cell simulation to a mismatch and shading effects of photovoltaic systems^18^,^19^,^20^. We developed a model of the PV cell array consisting of 8 identical cells interconnected in series via 7 isolation P3 scribes. The influence of the parallel resistance of the P3 laser scribe to the whole module efficiency was studied. The series resistance of the P2 interconnect was completely neglected in the calculations together with the P1 scribe resistance. The equivalent circuit of a mini-module is shown in Fig. 4. It is necessary to mention, that the equivalent circuit model and simulations were carried out using resistivity values (Ω∙m). However, in the section of the simulation results, we switch back to the conductivity (S/m) term in order to compare the results with the experimentally obtained values.

Each cell was implemented as a separate sub-circuit and is described by the following current-voltage relationship^21^:


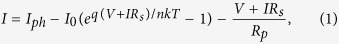


The photocurrent *I*_*ph*_ is generated when the cell is exposed to sunlight, *I*_*0*_ is the diode reverse saturation current caused by a minority carrier diffusion. Elementary charge is denoted as *q*, while *V* is the voltage across the output terminals of a solar cell. The series resistance *R*_*s*_ and the parallel resistance *R*_*p*_ affect the electrical current and the output voltage of a solar cell, respectively. The remaining parameters are the diode emission coefficient *n*, Boltzmann constant *k*, and *T* being the solar cell temperature.

The laser-induced effects can be incorporated into the *I-V* equation by considering that the solar cell’s parallel resistance comprises of shunt (*R*_*sh*_) and a laser scribe (*R*_*P*3_ = *ρ*/*L*) resistors connected in parallel:


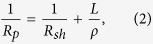


where *ρ* is the resistivity of the isolation scribe, *L* is the scribe length, and *R*_*sh*_ is the shunt resistance of a cell.

We simulated the operation of the 8-cell PV mini-module under the standard test conditions (STC). The parameters of the 13.1% efficiency solar cell module were taken from the electrical measurements of module 422_03. The laser scribe length was set to 4 cm, assuming, that the cell area was 0.4 × 4 cm^2^. Other considerations took into account that the serial interconnection does not cause any loss of the active area, or add the additional series resistance.

**Figure 5:**
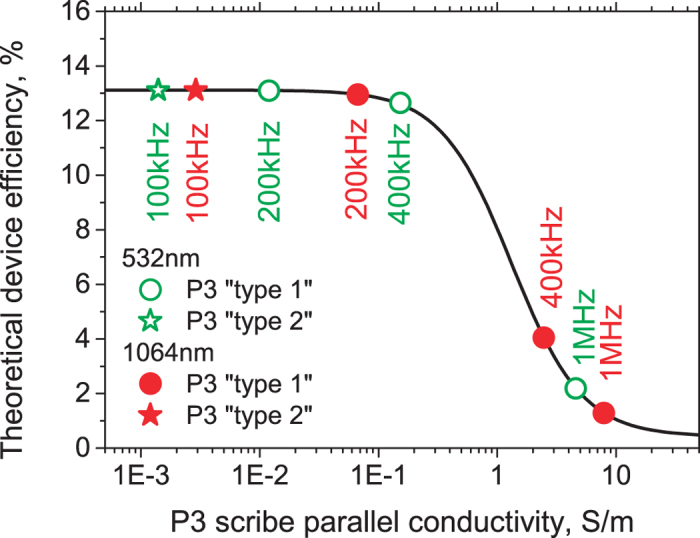
Theoretical device efficiency vs. P3 scribe conductivity. The simulation was performed for 8-cell mini-module. The solid red and hollow green dots represent 1064** **nm and 532** **nm wavelength patterning, respectively.

The simulation results achieved by using PSpice software shows a nonlinear solar module efficiency dependence on the conductivity of the isolation scribe (see Fig. 5). The high parallel conductance of the solar device induces alternative current paths for the generated photocurrent causing a partial short-circuiting of the device and reduction of its efficiency.

According to the simulations, the high-efficiency device should be processed with a P3 isolation scribe of less than 0.05 S/m conductivity. Otherwise, it can result in an efficiency drop more than 0.1% relative (see Fig. 5). In the case of the 1064 nm wavelength processing, the low repetition rate P3 “type 2” scribes with 100 kHz can offer low damage process with minor changes in the device efficiency. The 532 nm wavelength scribing can widen the process window up to the 100–200 kHz processing. However, the high-speed processing at a maximum laser repetition rate of 1 MHz results severe efficiency drop of 11.8 and 10.9% relative for the 1064 nm and 532 nm wavelengths, respectively (see Fig. 5). On the other hand, the P3 “type 2” 100 kHz processing via the top-contact layer lift-off can offer both – the high speed and low damage process. The changes of the mini-module efficiency were minimal for both 1064 nm and 532 nm wavelength patterning. Therefore, P3 “type 2” process is the most promising candidate for industrial applications.

### Ultra-high speed scribing

**Figure 6:**
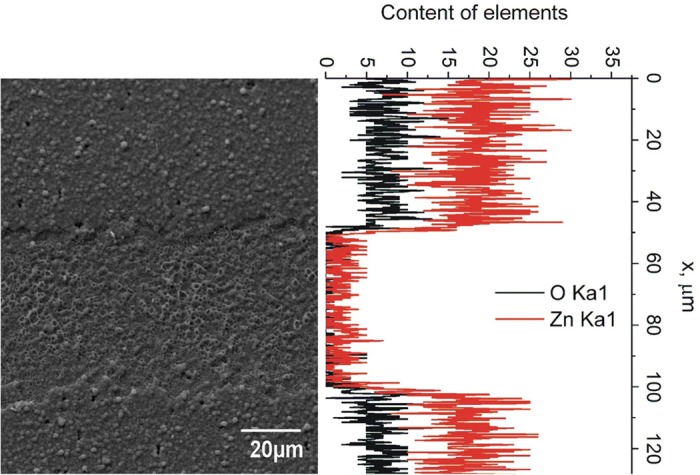
SEM image and the EDS analysis of the P3 laser scribe. Laser wavelength 1064** **nm, fluence 2.6** **J/cm^2^, repetition rate 1** **MHz, scribing speed 50** **m/s.

According to our investigations, the top-contact lift-off (P3 “type 2”) is the most promising process for cell isolation. Low pulse overlap is used in this case, therefore pushing the used galvanometer scanner to its speed limit. In our case, the 100 kHz laser pulse repetition rate was used, although the laser can support much higher rates up to 1 MHz. To push this process to the limit, the new beam scanning device was introduced. The Next Scan Technologies system does not use a set of galvoscanners but relies on rotating polygon scanner technology, capable of moving the focused laser spot at 25 to 100 m/s and higher speeds^22^. This technology enabled us to exploit the maximum available laser repetition rate for the P3 “type 2” processing setting the P3 scribing speed to 50 m/s. The SEM image together with the energy-dispersive X-ray spectroscopy (EDS) analysis are shown in Fig. 6. The EDS measurements confirmed the removal of the AZO top-contact leaving the underneath CIGS layer. Furthermore, the use of a low pulse overlap can significantly reduce the heat accumulation effects. In this case, the successive pulses are interacting with the laser-unaffected surface, therefore lowering the heat accumulation effect. That results in lower accumulated temperatures and higher ablation quality.

### Production of mini-modules

**Figure 7:**
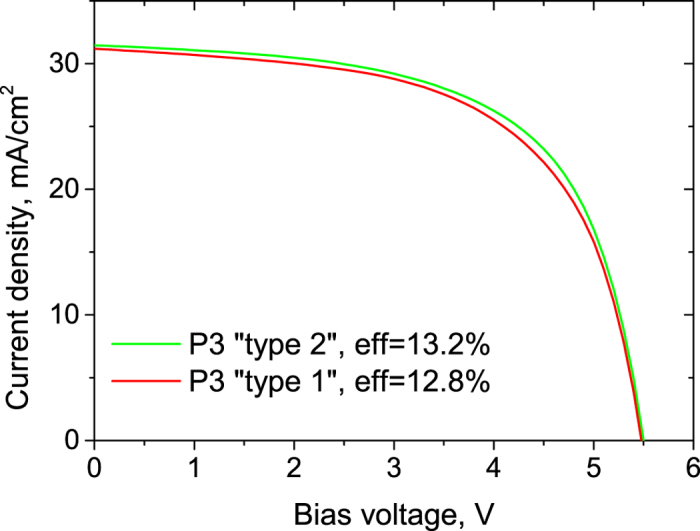
Module electrical performance. Two different P3 scribing approaches were used – direct laser ablation (P3 “type 1”) and top-contact lift-off (P3 “type 2”).

The superiority of laser scribed CIGS devices over the mechanical processing was already proved by several authors^23^. Furthermore, flexible CICS devices are difficult to structure by mechanical tools without damaging the soft polymer surface^24^. Therefore, all laser-scribed mini-modules were investigated in order to test the laser scribing process on a functional devices. The modules were produced on 50 × 50 mm^2^ float glass substrates. Each module consisted of 8 sub-cells. Each sub-cell was approximately 4 × 40 mm^2^. The devices were laser patterned at different stages of the production process. Already validated laser scribing processes for P1 and P2 patterns were applied. In both cases, the picosecond laser was used with wavelength of 532 nm. More details about these processes can be found in Table 2 and ref. 12. In the case of P3 patterning, two different scribing regimes were selected – removal of the CIGS layer to expose the Mo back-contact (P3 “type 1”) and removal of the front contact only (P3 “type 2”). The P3 channels were ablated using 532 nm wavelength, since it showed better scribe performance during the linear laser scribing technique (LLST) measurements. The parameters of the laser scribing processes are presented in Table 2. Unfortunately, lab-scale limitations did not allow us to perform high-speed P3 scribing of the mini-modules. Therefore, the laser repetition rates were reduced to 50 kHz for the P3 scribes. The results on the all laser-scribed mini-module performance are shown in the Fig. 7 and Table 3. The module efficiencies were measured just after the production. The antireflection coating was not introduced for both devices in this case.

The module 422_03 structured with P3 “type 2” regime showed better electrical performance. The efficiency and fill factor of particular device was 13.2% and 61.2%, respectively. The module 422_04 showed efficiency of 12.8% with fill factor of 59.9%. In this case, the P3 “type 1” pattern was used for the cell isolation. The direct laser ablation process resulted in formation of additional shunting paths near the scribing area, which reduced the fill factor and the efficiency of the module. The average individual cell shunt resistance values, extracted from the module *J-V* curves confirmed this assumption. The P3 “type 1” patterned cells showed shunt resistance of 297 Ω·cm^2^, while P3 “type 2” patterning resulted shunt resistance of 395 Ω·cm^2^. For both cases the series resistance of the module individual cells was very similar, therefore efficiency and fill factor was mainly influenced by the alterations in the shunt resistance of cells in the module. Although relatively low laser repetition rate of 50 kHz was used for both P3 “type 1” and “type 2” processing, the difference in module performance was observed. Nevertheless, the increase in the process throughput will require laser repetition rates of 1 MHz for P3 “type 1” process. According to the LLST measurements, this will result in even more severe shunting of the device and the reduction of efficiency. On the other hand, the P3 “type 2” process showed very low scribe conductivities at high speed scribing. Therefore it may be the only candidate for high volume module structuring.

## Summary

Classical approach to the P3 process (P3 “type 1”) includes scribing of the entire CIGS structure to expose the molybdenum back-contact^5^,^8^. Usually low power and pulse repetition rate lasers were used for scribing, resulting relatively low process speeds. On the other hand, this helped to avoid the thermal accumulation effects resulting satisfactory scribing quality. In the case of the industrial process implementation, high process throughput is required with an increase of the scribing speed, at least, up to 2 m/s. Fortunately, modern ultra-short pulsed lasers are powerful enough and can provide high pulse repetition rates. Therefore, the scribing speed could be linearly increased with the increase of the laser repetition rate keeping the optimal pulse overlap. We demonstrated that it is possible to upscale the P3 “type 1” patterning speed, although the thermal effects were more pronounced for high repetition rates. In the case of the 1064 nm wavelength processing, low repetition rate of 100 kHz offered high-quality scribing. Switching to 532 nm wavelength processing helped to increase the processing parameter window to 100–200 kHz. Exploitation of higher pulse repetition rates resulted in severe CIGS scribing quality decrease followed by electrical shunting of the device. On the other hand, high-speed top-contact layer lift-off (P3 “type 2” process) showed promising results. High scribing speed of 1.7 m/s was obtained at relatively low laser pulse repetition rate of 100 kHz. The P3 “type 2” scribe electrical testing indicated the parallel scribe conductivity of 0.0029 S/m for the 1064 nm wavelength processing. Use of the 532 nm wavelength helped to reduce the laser damage even further, resulting scribe conductivity of 0.0014 S/m. According to modelling, the laser induced P3 channel shunting of less than 0.05 S/m had no significant effect on the device performance. Furthermore, we pushed the P3 “type 2” process to the limit by applying a novel polygon scanning system. The scribing speed of 50 m/s was obtained exploiting the maximum available laser repetition rate of 1 MHz. Although high pulse repetition rate can lead to thermal damage, use of low pulse overlap processes can significantly reduce such effects. The all laser-scribed mini-module efficiency measurements also showed advantages of the low pulse overlap processing. The device, structured with low pulse overlap P3 “type 2” process showed efficiency of 13.2%. However, the reduction in CIGS device efficiency down to 12.8% was observed when the high pulse overlap P3 “type 1” process was used. This result was caused by parasitic shunting near the P3 “type 1” scribe area. Both LLST measurements and mini-module scribing test indicated, that low overlap P3 “type 2” scribing induced less cell shunting compared to high overlap P3 “type 1”. Taking all of this into account, it seems that the top-contact lift-off (P3 “type 2” process) is the only available solution for high-speed P3 patterning, which can be implemented in future TW production facilities.

## Materials and Methods

In this study, we focused on the P3 laser scribing process. The Atlantic series picosecond laser from Ekspla (pulse duration 13 ps, pulse repetition rate 1 MHz, wavelength 1064 nm/532 nm) was used for the scribing experiments. The experimental setup included the laser, beam expander and galvanometer scanner (ScanLab) with the focusing objective (focal length 80 mm) for each wavelength. In order to save a sample area, 0.7 mm laser scribes were applied setting the scribing speed limit of 1.7 m/s at these conditions. The minimum diffraction-limited spot size at the focus position was 22 μm for both wavelengths. In addition to the conventional galvanometer scanner, the polygon scanner (Next Scan Technology, LSE170 for the 1064 nm wavelength) was introduced extending the scanning speed range to 25–100 m/s. The focal length of the optical system was 190 mm with a spot size of 53 μm in this case. More details on the polygon system are provided by R. De Loor^22^.

In the case of the mini-module structuring, Onefive Katana 05 HP laser (pulse duration 50 ps, pulse repetition rate 0–100 MHz, wavelength 532 nm) combined with linear translation stages for mini-module positioning was used.

**Figure 8:**
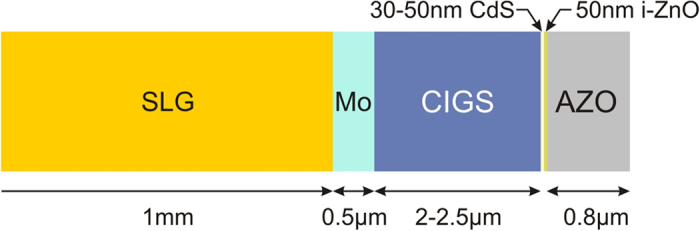
Structure of the CIGS solar cell. The sample was used in the laser scribing experiments.

Multilayer CIGS solar cell structures from EMPA with Al:ZnO (AZO) top-contact were used in the laser ablation experiments. The cells consisted of sputtered intrinsic ZnO and chemical-bath-deposited CdS buffer layers, thermally co-evaporated Cu(In,Ga)Se_2_ absorber layer, sputtered molybdenum back-contact and a soda lime glass (SLG) substrate. More details are provided in Fig. 8 and ref. 25.

**Figure 9:**
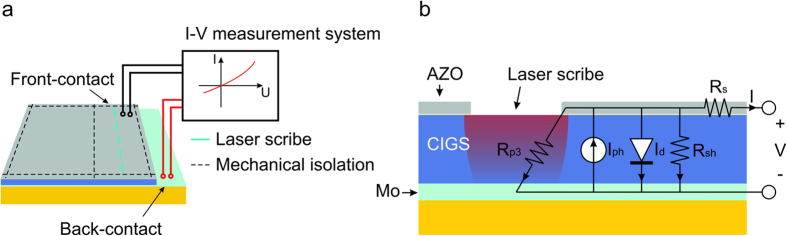
The setup of the linear laser scribing technique (LLST). (**a**) In-process parallel resistance measurement in fully functional mini-cell. (**b**) Typical equivalent circuit model of the laser scribed CIGS solar cell, where *R*_*s*_ is the serial resistance of a cell, *R*_*sh*_ is the parallel resistance of a cell, *R*_*p3*_ is the laser-induced parallel resistance, *I*_*d*_ is a diode current and *I*_ph_ is a photocurrent.

The laser processing with different laser pulse repetition rate and scribing speed was performed keeping the optimal pulse overlap. The quality of the scribes was evaluated visually by SEM (JEOL JSM-6490LV) together with energy-dispersive X-ray spectroscopy (EDS) followed by electrical characterization. Parallel resistance during the scribing process was assessed by applying 4 - point *I-V* measurements with Keithley (2602 A) *I-V* measurement system. We used linear laser scribing technique (LLST) to assess the scribe specific conductance. This method included laser scribing between the probe contacts (see Fig. 9a). Afterwards, the parallel resistance *R*_*sh*_ together with laser induced resistance *R*_*p3*_ was measured (see Fig. 9b). Then the scribing and cell resistance measurement process was repeated several times without contact probe removal. Then the data was converted to the conductance units. Finally, the laser scribe conductivity was extracted from the measurements by fitting the data with a simple linear function. More details on this method are provided in ref. 26.

## Additional Information

**How to cite this article**: Gečys, P. *et al*. CIGS thin-film solar module processing: case of high-speed laser scribing. *Sci. Rep.* **7**, 40502; doi: 10.1038/srep40502 (2017).

**Publisher's note:** Springer Nature remains neutral with regard to jurisdictional claims in published maps and institutional affiliations.

## Figures and Tables

**Table 1:** Laser processing parameters used in CIGS scribing.

No.	P3 process	Rep. rate, kHz	Fluence, J/cm^2^		Speed, m/s	Pulse overlap, %
			1064 nm	532 nm		
1	type 2	100	1.7	1.6	1.7	23
2	type 1	200	3.8	2.9	0.4	91
3	type 1	400	3.8	2.9	0.8	91
4	type 1	1000	3.8	2.9	1.7	92

**Table 2:** Laser processing parameters for mini-module scribing.

Module	P1	P2	P3 “type 1”	P3 “type 2”
422_03	Pulse overlap 40% Fluence 2.14 J/cm^2^	Pulse overlap 99% Fluence 0.32 J/cm^2^	—	Pulse overlap 30% Fluence 0.49 J/cm^2^
422_04	Pulse overlap 40% Fluence 2.14 J/cm^2^	Pulse overlap 99% Fluence 0.32 J/cm^2^	Pulse overlap 96% Fluence 0.65 J/cm^2^	—

**Table 3:** Module electrical properties.

Module	V_oc,_ V	J_sc_, mA/cm^2^	FF, %	Eff, %	R_s_, Ω·cm^2^ per cell	R_sh_, Ω·cm^2^ per cell	Process
422_03	5.50	31.3	61.2	13.2	2.84	395	P3 “type 2”
422_04	5.48	31.2	59.9	12.8	2.82	297	P3 “type 1”
